# Real-Time Spectroscopic Ellipsometry for Flux Calibrations in Multi-Source Co-Evaporation of Thin Films: Application to Rate Variations in CuInSe_2_ Deposition

**DOI:** 10.3390/ma17164048

**Published:** 2024-08-14

**Authors:** Dhurba R. Sapkota, Balaji Ramanujam, Puja Pradhan, Mohammed A. Razooqi Alaani, Ambalanath Shan, Michael J. Heben, Sylvain Marsillac, Nikolas J. Podraza, Robert W. Collins

**Affiliations:** 1 Wright Center for Photovoltaics Innovation & Commercialization, Department of Physics & Astronomy, University of Toledo, Toledo, OH 43606, USA; 2 Virginia Institute of Photovoltaics, Old Dominion University, Norfolk, VA 23529, USA

**Keywords:** multi-source co-evaporation, thin film deposition, thin film deposition calibration, real-time spectroscopic ellipsometry, film thickness, film deposition rate, film composition

## Abstract

Flux calibrations in multi-source thermal co-evaporation of thin films have been developed based on real-time spectroscopic ellipsometry (RTSE) measurements. This methodology has been applied to fabricate CuInSe_2_ (CIS) thin film photovoltaic (PV) absorbers, as an illustrative example, and their properties as functions of deposition rate have been studied. In this example, multiple Cu layers are deposited step-wise onto the same Si wafer substrate at different Cu evaporation source temperatures (*T*_Cu_). Multiple In_2_Se_3_ layers are deposited similarly at different In source temperatures (*T*_In_). Using RTSE, the Cu and In_2_Se_3_ deposition rates are determined as functions of *T*_Cu_ and *T*_In_. These rates, denoted *R*_eff_, are measured in terms of effective thickness which is the volume per planar substrate area and accounts for surface roughness variations with deposition time. By assuming that all incident metal atoms are incorporated into the films and that the atomic concentrations in the deposited material components are the same as in single crystals, initial estimates of the Cu and In atom fluxes can be made versus *T*_Cu_ and *T*_In_. Applying these estimates to the co-evaporation of a set of CIS films from individual Cu, In, and Se sources, atomic concentration corrections can be assigned to the Cu and In_2_Se_3_ calibration films. The corrections enable generation of a novel calibration diagram predicting the atomic ratio *y* = [Cu]/[In] and rate *R*_eff_ within the *T*_Cu_-*T*_In_ plane. Using this diagram, optimization of the CIS properties as a PV absorber can be achieved versus both *y* and *R*_eff_.

## 1. Introduction

Multi-source thermal co-evaporation as a deposition method for compound thin film materials is relatively straightforward in comparison with other widely applied methods such as magnetron sputtering and plasma-enhanced chemical vapor deposition (PECVD) [1,2,3]. In the co-evaporation method, the fluxes of atomic or molecular species incident on the film/substrate can be controlled accurately through the evaporation source temperatures, whereas in radio frequency (rf) sputtering and PECVD, the fluxes are controlled less directly through the rf electrical power applied to the target or cathode that generates the plasma. The sputtering or source-gas pressure, and even an applied target, cathode, or substrate dc bias, further affect the flux as additional deposition parameters that are used to moderate the average momentum per arriving atomic or molecular species at the film’s surface [4]. Thus, in co-evaporation, there exists a reduced number of parameters that require adjustment for optimization of the composition, structure, and electronic quality of the resulting compound. The properties of the materials deposited by co-evaporation then depend on the temperatures of the individual sources which control the atomic fluxes and, in turn, the deposition rate and material composition. The remaining deposition parameter is the substrate temperature which is the dominant means for controlling the film growth processes of adatom diffusion, nucleation, coalescence, and crystallite growth evolution [5,6,7].

Dimetal and trimetal chalcogenide thin films and their alloys are commonly prepared by multi-source co-evaporation, and the properties of these materials can be highly sensitive to the ratios of the incorporated metallic elements [8]. Of considerable technological interest among such chalcogenide thin films are the polycrystalline semiconductors having an average of four valence electrons per atom of the compound, yielding tetrahedral bonding. These semiconductors are designed on the basis of the Grimm–Sommerfeld rule starting from the II-VI binary compounds, extending to both ternary and quaternary compounds and their alloys [9]. In the deposition of such films by co-evaporation, the metal atom fluxes must be controlled accurately to ensure the desired atomic ratios, whereas a high flux of the group VI element, along with an elevated growth temperature, must be used to ensure the desired phase, microstructure, and crystallinity. Examples of the thin film semiconductor materials having photovoltaics applications include the ternary compound CuInSe_2_ (CIS) along with the wider bandgap alloys of CIS with CuGaSe_2_ (CIGS) and CuAlSe_2_ (CIAS) [10,11,12,13], and the quaternary compound Cu_2_ZnSnSe_4_ (CZTSe), along with the wider bandgap alloy of CZTSe with Cu_2_ZnSnS_4_ (CZTSSe) [14].

For these materials, electron impact emission spectroscopy (EIES) can be adopted as an analytical method for monitoring and controlling the atomic fluxes of the individual metallic elements and, thus, the composition of the resulting compounds and alloys [15,16]. The EIES outputs can be calibrated in terms of atomic fluxes on the basis of mass/area deposition rates determined using a quartz crystal oscillator moved to the substrate location of interest. As an alternative analytical method for flux determination, real-time spectroscopic ellipsometry (RTSE) can be applied to provide a direct measurement of the condensed film surface (as opposed to the atomic vapor) at the specific location of interest, yielding rapid identification of the film volume/area or effective thickness at that location through optical modeling of the RTSE spectra. In addition to the effective thickness of the film, the RTSE measurement provides the advantage of optical property and bandgap determination of the material from the same optical modeling procedure. This latter capability enables more direct determination of the compositions of compounds and alloys from the film surface itself [17,18]. 

In this study, an RTSE methodology for calibration of the metal atom fluxes is presented in detail, and its application to the deposition rate variation of the dimetal chalcopyrite CIS is described as an example. Potential applications exist to other dimetal chalcogenide compounds and alloys, as well as to the trimetals. The calibration concept was first developed to assist in the fabrication of one-stage and two-stage CIS absorbers at different deposition rates for incorporation into solar cells [19,20]. Here the detailed procedures toward further evaluation and implementation of the methodology are presented. 

As the first step of the methodology, a succession of Cu thin films, each with a different Cu evaporation source temperature, was deposited on a crystalline silicon (c-Si) substrate and monitored by RTSE to establish the effective thickness rate (or volume per planar substrate area) as a function of the source temperature. A similar experiment was performed for In_2_Se_3_ thin films. Although a more straightforward approach would be to calibrate the In source by RTSE during deposition of elemental In, rather than In_2_Se_3_, the morphological evolution during In deposition was found to be too extreme for accurate RTSE analyses. In fact, a bulk layer of In does not develop during the full range of semi-transparency due to the evolution of isolated In particles. Furthermore, given the deposition of In_2_Se_3_ for calibration of the In source, it would seem possible to calibrate the Cu source by deposition of Cu_2−*x*_Se of known *x*. For Cu_2−*x*_Se, however, the surface roughness layer was found to be greater in thickness than the bulk layer during the range of semi-transparency. Thus, as in the case of metallic In, Cu_2−*x*_Se is also less suitable for accurate calibration.

As the second step, the atomic fluxes of Cu and In can be estimated as functions of the source temperatures based on the assumptions of unity metal atom sticking coefficient in the deposition of the Cu and In_2_Se_3_ calibration films, as well as single crystal atomic concentrations in the deposited component materials. Adopting the corresponding assumptions for CIS deposition, the resulting calibration can be presented in a novel graphical form and applied in a third step to predict the effective thickness rates and compositions of CIS thin films deposited with specified Cu and In source temperatures. Finally, the deviations of the measured CIS rates and compositions from these predictions provide atomic concentration corrections that can be assigned to the calibration depositions. With these corrections, the Cu and In source temperatures that generate CIS films of accurately specified deposition rate and composition for optimization of the film properties can be determined. Applying the calibration developed in this study, CIS films with *y* = [Cu]/[In] values fixed in the range of 0.90 ± 0.03 can be deposited, spanning the range of effective thickness rates from 3 Å/s to 8 Å/s. Increases in both the crystallite size and the steepness of the Urbach tail have been observed with decreasing deposition rates down to the lowest explored value of ~3 Å/s. This suggests improved photoelectronic properties for the lowest deposition rate materials, made possible by the calibration procedure developed in this study.

## 2. Materials and Methods

**Figure 1:**
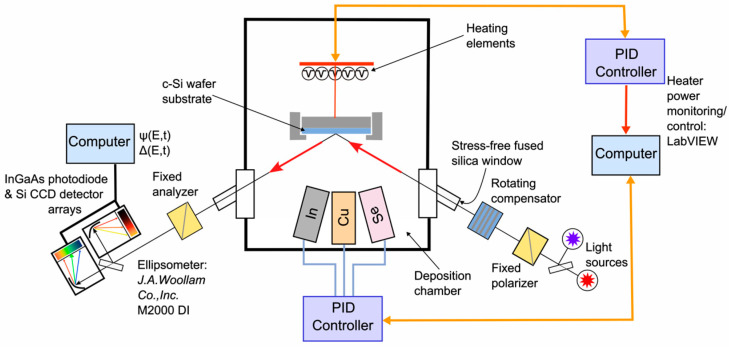
Schematic diagram of a co-evaporation chamber used for CuInSe_2_ deposition equipped with a thin film growth analysis capability by rotating compensator real-time spectroscopic ellipsometry. The diagram also illustrates the components of the ellipsometer on the polarization-generation arm on the right and the polarization-detection arm on the left.

Figure 1 shows the experimental configuration for CIS deposition using a multi-source thermal co-evaporation system that incorporates instrumentation for RTSE measurement and monitoring. In the calibration experiments performed here, Cu and In_2_Se_3_ films were deposited step-wise on native oxide-covered c-Si wafers at room temperature and at 570 °C, respectively, using Cu and In evaporation sources held at different temperatures *T*_Cu_ and *T*_In_. The sources incorporate alumina crucibles and Mo crucible liners (Radak II, Luxel, Friday Harbor, WA, USA). For the Cu and In_2_Se_3_ calibration depositions, the source temperatures spanned the ranges of *T*_Cu_ = 1335–1395 °C and *T*_In_ = 965–1025 °C, as used in the deposition of CIS thin films. These temperature ranges yield effective thickness rates of Cu and In_2_Se_3_ within the ranges of 0.35–1.03 Å/s and 2.83–6.76 Å/s, respectively.

Thin film CIS materials and CIS solar cell absorbers for devices were deposited in thicknesses within the range of 1.5–2.0 μm at different rates for evaluation of the calibration and for measurement of the CIS film properties as functions of the deposition rate. These depositions were performed over similar ranges as the calibrations, *T*_Cu_ = 1335–1385 °C and *T*_In_ = 955–1025 °C. For both In_2_Se_3_ and CIS thin films, a constant flux of Se leading to a Se film growth rate of 20 Å/s was maintained throughout the depositions. The growth rate of Se was estimated by a room temperature quartz crystal monitor at the substrate position in the absence of In and Cu flux. The source temperature and rate ranges for CIS deposition were limited at low temperatures by the stability of the fluxes and at high temperatures by the capacity of the sources, given the time required to reach stability at the given rate and then to deposit a 2 μm-thick CIS film.

For the RTSE measurements, a rotating compensator multichannel spectroscopic ellipsometer with a photon energy range of 0.75 to 6.5 eV was used (Model M2000-DI, J. A. Woollam Co., Inc, Lincoln, NE, USA). For each RTSE measurement of the (ψ, Δ) spectra, the complete data cycle occurred over a time of 2.6 s. The ellipsometry parameters (tanψ, Δ) were the *p*/*s* ratio of relative complex optical electric field amplitudes (reflected relative to incident) and the *p − s* difference in the field phase shifts (upon reflection), respectively. In the 2.6 s cycle time, data were collected over a 1 s time period at an analyzer angle of *A* = ±45°, the analyzer was rotated to *−A* over a period of 0.6 s, and data were collected again over a 1 s time period at the second analyzer angle. In each 1 s data collection, results from a total of 39 mechanical cycles of the rotating compensator (or 78 optical cycles) were averaged to enhance precision. In addition, the results for the two 1 s acquisitions at ±*A* were averaged to enhance accuracy. As a result, the final (ψ, Δ) spectra represent an average over 156 optical cycles of the rotating compensator. In the context of RTSE, the thickness resolution describes the film thickness that was deposited in a single data cycle time. At the minimum and maximum effective thickness rates for the Cu calibration deposition, monolayer scale thickness resolutions of 0.9 Å and 2.7 Å, respectively, were achieved. For the In_2_Se_3_ calibration deposition, the corresponding thickness resolutions are poorer, 7.4 Å and 17.6 Å, respectively.

The atomic ratios *y* = [Cu]/[In] of the CIS films in this study were obtained by energy-dispersive X-ray spectroscopy (EDS). The EDS measurements were performed using a tabletop microscope fitted with an EDS unit (Hitachi TM-1000, Oxford Instruments, Abingdon, UK). Estimates of the crystalline grain sizes of the films were obtained from X-ray diffraction (XRD) measurements with a small-angle X-ray scattering (SAXS) capability (Ultima II, Rigaku, Tokyo, Japan).

## 3. Results

### 3.1. Real-Time Spectroscopic Ellipsometry: Cu and In_2_Se_3_ Calibration Depositions

**Figure 2:**
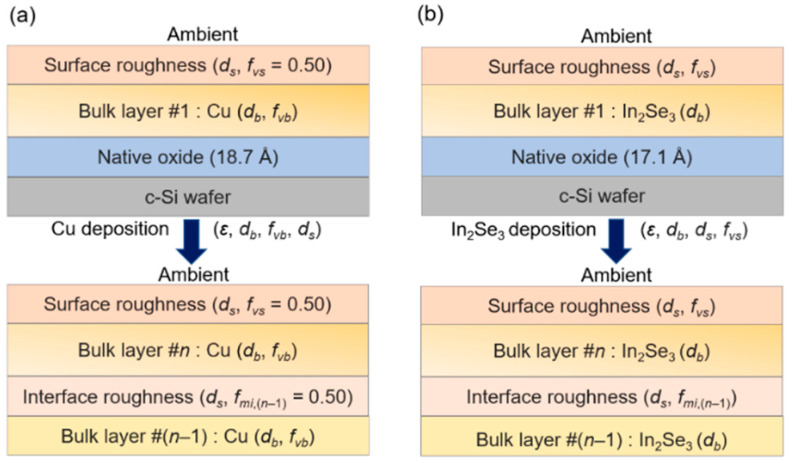
Structural models used in the analysis of real-time spectroscopic ellipsometry data acquired during the first steps (upper panels) and the *n*th steps (lower panels) of the (**a**) Cu and (**b**) In_2_Se_3_ depositions performed for Cu and In evaporation source calibrations, respectively. For the lower two panels, only the top-most *n*th (*n* > 1) layer and its underlying (*n* − 1)st layer are shown. The depositions were performed step-wise using different source temperatures on native oxide-coated crystalline silicon substrates at room temperature for Cu and 570 °C for In_2_Se_3_. The variable structural parameters in the models include the bulk and surface roughness layer thicknesses *d_b_* and *d_s_*. For the surface and interface roughness layers of the Cu depositions, 0.50/0.50 volume fraction composites of the underlying/overlying media are used, and for the respective layers of In_2_Se_3_ (1 − *f_vs_*)/*f_vs_* and *f_mi_*_,(*n*−1)_/*f_mi_*_,*n*_, composites are used. The bulk layer void content *f_vb_* for Cu is determined from the Drude component of the complex dielectric function ε over the photon energy range of 0.75–1.00 eV. For the first step-wise Cu layer, *f_vb_* is assumed to be the same as that of the second layer.

For evaporation source calibrations, the effective thickness evolution of Cu thin films was obtained by RTSE analysis of a step-wise deposition process on a single room temperature c-Si wafer substrate with each step being performed at a different Cu evaporation source temperature. The deposition time per step was ~1 min, selected in order to avoid opacity of the accumulated film structure after a total of five deposition steps. As a result, accuracy in the determination of the effective thickness was maintained, even in the fifth step when the film was the thickest. Figure 2a (top) shows the structural model used in the RTSE analysis of the first step of the Cu calibration deposition. This model includes a native oxide layer on the c-Si substrate, a Cu bulk layer, and a Cu surface roughness layer, the latter having a complex dielectric function spectrum ε, modeled using the Bruggeman effective medium approximation (EMA) as a 0.5/0.5 volume fraction mixture of the bulk layer Cu and void [21]. The Si oxide layer thickness is shown as 18.7 Å in Figure 2a (top) and was determined from an analysis of the in situ SE spectra acquired before the step-wise deposition process. 

The complex ε spectrum of the Cu bulk layer for each deposition step was determined in a multi-time analysis procedure using the ten (ψ, Δ) spectra acquired over the final 26 s of the ~1 min deposition time period. For the steps of the analysis after the first, the previous steps served to generate the substrate structure which, in contrast to the first step, included a surface roughness layer, as shown in Figure 2a (bottom). This schematic below shows only the topmost bulk/roughness layers and their underlying two layers. For simplicity, the data collected during the short initial time period of each step after the first, corresponding to simultaneous interface-filling and new roughness-layer development, were not analyzed in the determination of the effective thickness rate. Completion of interface-filling yields an interface-roughness layer with a *f_mi_*_,(*n*−1)_/*f_mi_*_,*n*_ = 0.5/0.5 mixture of the underlying (*n* − 1) material and overlying (*n*) material of the *n*^th^ deposition step. Thus, the (ψ, Δ) spectra considered here were confined to those collected after the interface layer was completely filled, meaning that *f_mi_*_,*n*_ could be fixed at 0.5.

The complex ε spectrum of the Cu bulk layer for each deposition step was fitted over the low photon energy range from 0.75 to 1.0 eV using a single Drude term with variable resistivity ρ and scattering time τ, as well as a variable photon energy independent real contribution to ε. In estimating the void volume fraction of the Cu bulk layer, the electron effective mass was fixed as m_e_* = 1.01*m_e_*, where *m_e_* is the free electron mass [22]. The values of ρ, τ, and m_e_*, along with the electron charge *e*, provide the electron concentration *N_e_* via the equation *N_e_* = m_e_*/*e*^2^ρτ, which is also equated to the atomic concentration *N_a_* assuming a unity valence. The void volume fraction in the Cu bulk layer is then estimated as *f_vb_* = 1 − (*N_a_*/*N_ac_*), where *N_ac_* is the concentration of atoms in single-crystal Cu, determined from the room temperature lattice parameter of *a* = 3.6147 Å [23]. Any inaccuracies in calibration as a result of this procedure can be compensated for by application of the atomic concentration corrections to be described in Section 3.4.

**Figure 3:**
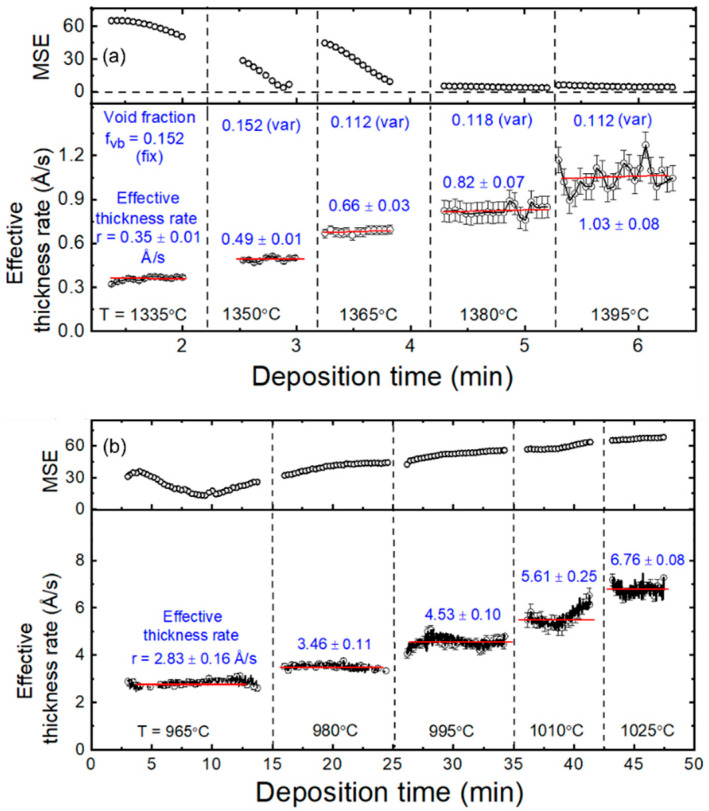
Real-time spectroscopic ellipsometry (RTSE) analysis of (**a**) Cu and (**b**) In_2_Se_3_ thin films deposited step-wise by evaporation on c-Si substrates at room temperature and at 570 °C, respectively, using five different Cu and In source temperatures, as demarcated by the vertical broken lines. Shown in each top panel is the mean square error (MSE) from the best fit of the RTSE data, and in each bottom panel the instantaneous effective thickness deposition rate *R*_eff_ = d*d*_eff_/d*t*, which is the instantaneous deposition rate in terms of material volume/area. Average values of the effective thickness deposition rate are included at each source temperature step. For Cu, values of the bulk layer void volume fraction *f_vb_* at each temperature step are also included, as deduced from an analysis of the Drude components of the complex dielectric functions ε over the photon energy range of 0.75–1.00 eV.

Once the ε spectrum spanning the full photon energy range of 0.75 to 6.5 eV is available for the Cu bulk layer at each step, the structural evolution versus the accumulated deposition time can be obtained. The details are described in Reference [19] and the final results of interest here are shown in Figure 3a. Here, the vertical broken lines indicate the increases in source temperature during the continuous step-wise deposition process occurring on the same substrate. To ensure stable deposition rates, a shutter was closed in order to block deposition on the substrate for a 30 min time period during which the new source temperature was set and the Cu flux was allowed to stabilize at the new temperature. After this time period, the shutter was opened for deposition, and RTSE data were collected for the given fixed source temperature. Thus, the horizontal axis includes only the elapsed time during which the shutter was open for Cu film deposition.

In the RTSE analysis results of Figure 3a for the Cu source calibration, the upper panel depicts the time evolution of the mean square error (MSE), which is a measure of the quality of the fit, and the lower panel depicts the instantaneous effective thickness deposition rate *R*_eff_ = d*d*_eff_/d*t*. The effective thickness *d*_eff_ is the volume per planar area of the substrate and is given in this case (disregarding the time-independent interface contribution) as *d*_eff_ = (1 − *f_vb_*)*d_b_* + (1 − *f_vs_*) *d_s_* = (1 − *f_vb_*) *d_b_* + 0.5*d_s_*, where *f_vb_* and *f_vs_* are the void volume fractions in the bulk and surface roughness layers with *f_vs_* = 0.50 [19]. The panel of Figure 3a depicting the effective thickness rate also indicates the results of the Drude analysis of ε, yielding *f_vb_* values in the range of 0.11 to 0.15. The ability to extract bulk layer void fractions by this method is reduced for the first deposition step at the lowest Cu source temperature of 1335 °C due to the thinness of the Cu layer. As a result, *f_vb_* for this first step is fixed at the value obtained for the second step of 1350 °C. The results in Figure 3a suggest that the flux of Cu is stable over the time periods of 0.5 to 1 min at each deposition temperature.

For calibration of the In evaporation source, the structural evolution for step-wise In_2_Se_3_ thin films was obtained by RTSE data analysis over five deposition steps in a procedure similar to that described for Cu [19]. The structural model is depicted in Figure 2b, and the analysis results for the instantaneous effective thickness deposition rate are presented in Figure 3b. The time for each deposition step at a given source temperature was 5–15 min in this case, longer than that for Cu since the In_2_Se_3_ was semitransparent below its bandgap of *E_g_* ≈ 2.2 eV, even for very thick layers [24]. For the In_2_Se_3_ deposition process as well, a shutter was closed in order to block the substrate for a 30 min time period, during which the In source temperature was increased between deposition steps and the In flux was allowed to stabilize at the source temperature setting. RTSE analyses of In_2_Se_3_ involved a multi-time method for determination of the complex ε spectrum over each step, which was then applied to determine the structural evolution throughout the film growth. In contrast to the Cu step-wise deposition, no reliable method is available for determining an absolute bulk layer void volume fraction (i.e., relative to the zero value of the ideal single crystal) from the complex ε spectrum for each of the deposited In_2_Se_3_ layers. Also in contrast to the Cu deposition, the In_2_Se_3_ deposition in Figure 3b shows time variations in the effective thickness rates. A possible origin of these variations is the longer time scale of each deposition step and the variations in the In evaporation source power needed to maintain the constant source temperature as the source contents become depleted.

### 3.2. Cu and In Flux Analysis

**Figure 4:**
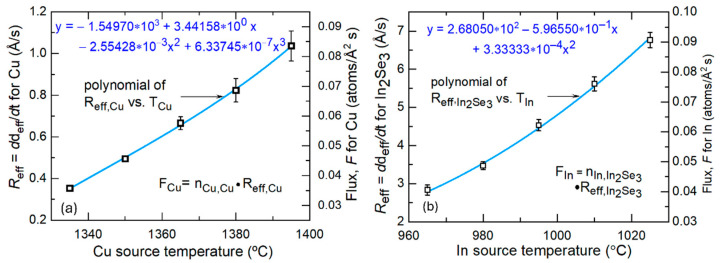
Deposition rate in terms of effective thickness from the real-time spectroscopic ellipsometry (RTSE) data of Figure 3a,b for (**a**) Cu and (**b**) In_2_Se_3_, plotted versus the Cu and In evaporation source temperatures, respectively. The RTSE data were collected during the step-wise deposition of five successive layers on a c-Si wafer at room substrate temperature for Cu and at 570 °C for In_2_Se_3_. Also shown on the right-hand scale are the Cu and In atom fluxes calculated from the effective thickness rates based on the assumptions of Cu calibration depositions with bulk material void fractions in the 0.112–0.152 range, implicitly included in the effective thickness rates as in Figure 3a, and In_2_Se_3_ depositions with single crystal density.

Figure 4 presents the deposition rates of Cu and In_2_Se_3_, given in terms of effective thicknesses from Figure 3a,b, plotted as functions of the respective Cu and In source temperatures. Also shown in Figure 4 are the two best fitting polynomials that describe the relationships. The next step in the calibration analysis is to derive the fluxes of the Cu and In atoms, *F_x_* = (*F*_Cu_, *F*_In_) as functions of the effective thickness deposition rates of Cu and In_2_Se_3_, *R*_eff,*x*,*m*_ = (*R*_eff,Cu,Cu_, *R*_eff,In,In_2_Se_3__), respectively, where the subscript *x* indicates the metallic element and *m* indicates the deposited material. In a general film growth process, the atomic flux of element *x* can be evaluated from the effective thickness rate of material *m* by assuming that all evaporated atoms impinging on the substrate are incorporated into the growing film. As a result, the flux of atom *x* is given by

(1) $$F_{x}=\left(1-f_{v,m}\right)\;n_{x,c\text{-}m}R_{\mathrm{eff},x,m\;}$$

where *n_x_*_,c-*m*_ represents the concentration of atoms *x* in the single crystal of material *m*, and *f_v,m_* is any material deficit or void volume fraction in the thin film material, *m*, relative to the single crystal that is not accounted for through the rate *R*_eff,*x*,*m*_. If such unaccounted-for voids (i.e., an excess or deficit) exist, then the factor of 1 − *f_v_*_,*m*_ must be introduced for proper determination of *F_x_* since the concentration of atom *x* in the material *m* is altered relative to the assumed value of the single crystal. The use of a single factor 1 − *f_v_*_,*m*_ in Equation (1) is further based on the assumption that the excess or deficit void volume fraction *f_v_*_,*m*_ exists not only in the bulk layer but also in the components of the surface roughness layer and any interface roughness layer that may also be included in the effective thickness.

Presented in Table 1 are literature values of the atomic concentrations of Cu *n*_Cu,c-Cu_ and In *n*_In,c-In2Se3_ in the single crystals of Cu and In_2_Se_3_ at the deposition temperatures of room temperature and 570 °C, respectively, for application in Equation (1). Table 1 also provides the references from which these data are drawn [23,25,26]. Considering the factor 1 − *f_v_*_,*m*_ in Equation (1) for the case of the Cu calibration deposition, a void content was accounted for through *R*_eff,Cu,Cu_ by estimating the electron concentration from the Drude term of the ε spectrum and equating it to the atomic concentration. In spite of this estimate, however, an additional non-zero void fraction *f_v_*_,Cu_ in Equation (1) may be needed in order to establish an accurate Cu deposition flux. As a result, an excess or deficit relative to that estimated on the basis of the Drude analysis is introduced. In contrast, in the case of In_2_Se_3_, no RTSE analysis method is available to estimate the void content of the bulk material relative to the single crystal and to incorporate it into *R*_eff,In,In2Se3_. Thus, in this case, it is even more likely that *f_v_*,_In2Se3_ is non-zero due to unaccounted-for voids in grain boundary regions or tensile strains in crystallites, generating a density deficit (*f_v_*_,In2Se3_ > 0) and/or due to compressive strains, generating a density excess (*f_v_*_,In2Se3_ < 0). 

For a workable CIS calibration as described in the next section, it is necessary to express the two source temperatures *T_x_* (*x* = Cu, In) first as polynomial functions of the Cu and In_2_Se_3_ effective thickness deposition rates *R*_eff,*x*,*m*_, i.e., the inversions of the polynomial functions in Figure 4, and then as polynomial functions of the Cu and In atom fluxes *F_x_* applying Equation (1), which is solved for *R*_eff,*x*,*m*_. For this purpose, closely fitting results for *T_x_* as functions of *R*_eff,*x*,*m*_ can be obtained using third-order polynomials with the coefficients *c_m_*_,*n*_; *n* = 0–3, introduced in Table 2. Given the Cu and In_2_Se_3_ single-crystal structures of Table 1 and any additional void fractions, the source temperatures can be expressed in turn as third-order polynomials in the Cu and In fluxes with *n*th-order coefficients given by *d_m_*.*_n_* = *c_m_*_,*n*_/{(1 − *f_v_*_,*m*_) *n_x_*._c-*m*_}*^n^*. The coefficients *d_m_*_,*n*_; *n* = 0–3 are also presented in Table 2, assuming at this stage that *f_v_*_,*m*_ = 0. Thus, the defining equations for these latter coefficients are as follows:

(2) $$\begin{array}{cc} T_{\mathrm{Cu}} & =\overset{3}{\underset{n=0}{\sum }}c_{\mathrm{Cu},n}{\left(R_{\mathrm{eff},\mathrm{Cu},\mathrm{Cu}}\right)}^{n}\\ & =\sum_{n=0}^{3}\frac{c_{\mathrm{Cu},n}}{{\left\{\left(1-f_{v,\mathrm{Cu}}\right)n_{\mathrm{Cu},c\text{-}\mathrm{Cu}}\right\}}^{n}}{F_{\mathrm{Cu}}}^{n}=\sum_{n=0}^{3}d_{\mathrm{Cu},n}{F_{\mathrm{Cu}}}^{n} \end{array}$$

(3) $$\begin{array}{cc} T_{\mathrm{In}} & =\overset{3}{\underset{n=0}{\sum }}c_{{\mathrm{In}}_{2}{\mathrm{Se}}_{3},n}{\left(R_{\mathrm{eff},\mathrm{In},{\mathrm{In}}_{2}{\mathrm{Se}}_{3}}\right)}^{n}\\ & =\sum_{n=0}^{3}\frac{c_{{\mathrm{In}}_{2}{\mathrm{Se}}_{3},n}}{{\left\{\left(1-f_{v,{\mathrm{In}}_{2}{\mathrm{Se}}_{3}}\right)n_{\mathrm{In},c\text{-}{\mathrm{In}}_{2}{\mathrm{Se}}_{3}}\right\}}^{n}}{F_{\mathrm{In}}}^{n}=\sum_{n=0}^{3}d_{{\mathrm{In}}_{2}{\mathrm{Se}}_{3},n}{F_{\mathrm{In}}}^{n} \end{array}$$

Thus, the second equalities in each of these two equations are determined from the general Equation (1), solving for the effective thickness rates *R*_eff,*x*,*m*_ in terms of the fluxes *F_x_* and applying the result to Cu (with subscripts *x*: Cu, *m*: Cu) and to In (*x*: In, *m*: In_2_Se_3_).

### 3.3. Development of Source Calibration for CIS Deposition

The focus turns next to the calibration of the CIS deposition in which the Cu and In sources are operated simultaneously so as to obtain a desired Cu-to-In atomic ratio *y*, typically *y* ~ 0.9 for photovoltaic applications [12,13]. To achieve this result, the Cu and In atomic fluxes must be accurately determined, and their ratio set according to *F*_Cu_/*F*_In_ = *y*, based on the assumption that all metal atoms impinging on the substrate are incorporated into the CIS film. Subsequent calibration for the CIS effective thickness deposition rate *R*_eff,CIS_, given the desired *y* value, is of interest in order to optimize the material within the available parameter space, which typically includes not only the flux ratios and substrate temperature, but also the CIS deposition rate. The polynomials in *F*_Cu_ and *F*_In_ of Equations (2) and (3), with the coefficients *d_m_._n_*, can be expressed in terms of the CIS effective thickness rate *R*_eff,CIS_ by applying the CIS analogs of Equation (1). Thus, applying *F*_In_ = (1 − *f_v_*_,CIS_) *n*_In,CIS_ *R*_eff,CIS_ to Equations (2) and (3) with *n*_Cu,c-CIS_ = *y n*_In,c-CIS_ and *f_v_*_,CIS_ as the void volume fraction within the deposited CIS thin films, yields

(4) $$\begin{array}{cc} T_{\mathrm{Cu}} & =\overset{3}{\underset{n=0}{\sum }}\frac{c_{\mathrm{Cu},n}}{{\left\{\left(1-f_{v,\mathrm{Cu}}\right)n_{\mathrm{Cu},c\text{-}\mathrm{Cu}}\right\}}^{n}}{\left\{y\left(1-f_{v,\mathrm{CIS}}\right)n_{\mathrm{In},c\text{-}\mathrm{CIS}}R_{\mathrm{eff},\mathrm{CIS}}\right\}}^{n}\\ & =\sum_{n=0}^{3}d_{\mathrm{Cu},n}{\left\{y\left(1-f_{v,\mathrm{CIS}}\right)n_{\mathrm{In},c\text{-}\mathrm{CIS}}R_{\mathrm{eff},\mathrm{CIS}}\right\}}^{n} \end{array}$$

(5) $$\begin{array}{cc} T_{\mathrm{In}} & =\overset{3}{\underset{n=0}{\sum }}\frac{c_{{\mathrm{In}}_{2}{\mathrm{Se}}_{3},n}}{{\left\{\left(1-f_{v,{\mathrm{In}}_{2}{\mathrm{Se}}_{3}}\right)n_{\mathrm{In},c\text{-}{\mathrm{In}}_{2}{\mathrm{Se}}_{3}}\right\}}^{n}}{\left\{\left(1-f_{v,\mathrm{CIS}}\right)n_{\mathrm{In},c\text{-}\mathrm{CIS}}R_{\mathrm{eff},\mathrm{CIS}}\right\}}^{n}\\ & =\sum_{n=0}^{3}d_{{\mathrm{In}}_{2}{\mathrm{Se}}_{3},n}{\left\{\left(1-f_{v,\mathrm{CIS}}\right)n_{\mathrm{In},c\text{-}\mathrm{CIS}}\;R_{\mathrm{eff},\mathrm{CIS}}\right\}}^{n} \end{array}$$

Application of the data in Table 1, including the relation between the Cu and In concentrations in CIS, is based on a model in which Cu poor CIS (*y* < 1) is obtained from stoichiometric single-crystal CIS [27,28] via introduction of uncompensated Cu vacancies without a change in lattice constants upon reduction in *y*. Such a model is over-simplified as it does not account for the observed polycrystallinity and stress in the thin films, or for their low hole concentrations [12,13,29]. Together with the assumptions that *f_v_*_,*m*_ = 0 for *m* = Cu, In_2_Se_3_, and CIS, however, the model is a reasonable starting point for the calibration.

**Figure 5:**
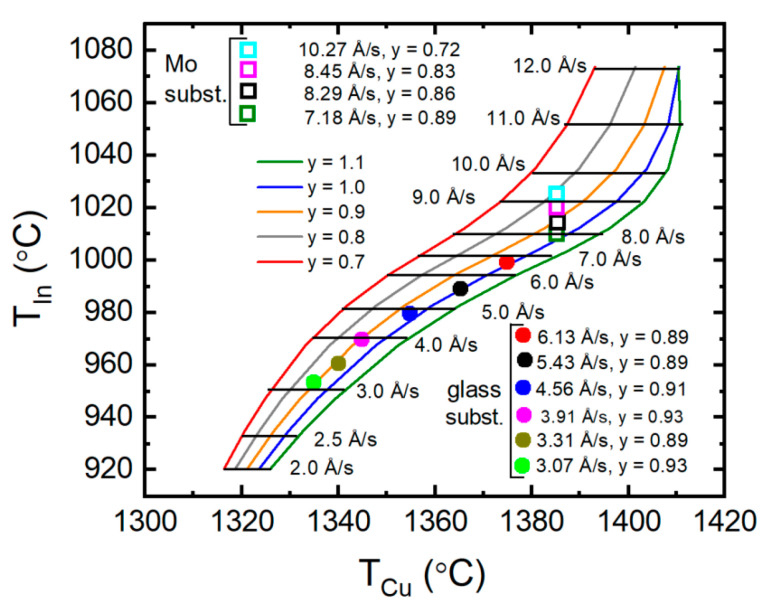
Calibration curves for the settings of the In and Cu source temperatures in CuInSe_2_ deposition required to obtain specific values of the CIS deposition rate *R*_eff,CIS_ (horizontal lines) and the [Cu]/[In] composition ratio *y* (curves). The calibration curves were calculated by applying Equations (4) and (5) using the coefficients *d_m_*_,*n*_ of Table 2 obtained from the Cu and In_2_Se_3_ calibration depositions of Figure 4. Experimental results for comparison are included from two series of depositions, one series of CIS films of different *y* on Mo-coated glass substrates (open squares) and a second series of CIS absorber layer witness samples deposited directly on glass with intended *y* = 0.90 at different deposition rates *R*_eff,CIS_ for solar cells (solid circles). Calculations are based on the assumptions of (i) thin film Cu depositions with void fractions of 0.112–0.152, implicitly included in *R*_eff,Cu,Cu_ as in Figure 3a, and (ii) thicker In_2_Se_3_ and CIS depositions having single-crystal density (*f_v_*_,In2Se3_ = *f_v_*_,CIS_ = 0).

Equations (4) and (5) can provide the two source temperatures required to achieve specified values of the CIS effective thickness deposition rate *R*_eff,CIS_ and [Cu]/[In] ratio *y*. These calculations apply (i) the polynomial coefficients *d_m_*_,*n*_ of Table 2, relevant for the Cu and In fluxes, assuming *f_v_*_,Cu_ and *f_v_*_,In2Se3_ both vanish, and (ii) the concentration of In in CIS from Table 1, applying the model as described in the previous paragraph assuming *f_v_*_,CIS_ = 0. Thus, (*R*_eff,CIS_, *y*) are substituted into the right sides of these equations to give the pair of source temperatures (*T*_Cu_, *T*_In_). Deposition rate values *R*_eff,CIS_ in terms of effective thickness ranging from 2 Å/s to 12 Å/s were assumed for five different *y* values centered at *y* = 0.9, including *y* = 0.7, 0.8, 0.9, 1.0, and 1.1 (with *y* > 1 representing Cu-rich CIS assuming interstitial Cu atoms). The resulting pairs of source temperatures (*T*_Cu_, *T*_In_) are plotted as (abscissa, ordinate) in Figure 5 to depict the starting point calibration for CIS deposition. Horizontal lines at constant *T*_In_ identify equal CIS deposition rates, and the curves identify equal CIS [Cu]/[In] atomic ratios.

For evaluation of the validity of the starting point calibration and the model with the several assumptions on which it is based, two sets of experimental results are plotted in Figure 5. One series of samples with thicknesses within the range ~1.5–2.0 µm was prepared directly on Mo-coated glass substrates with fixed Cu source temperature and variable In source temperature in an attempt to span the range of *y* from 0.8 to 1.0 based on trial and error, albeit with a variable deposition rate *R*_eff,CIS_ due to the variable *T*_In_. RTSE analysis of the initial ~700 Å film thickness was performed to obtain the deposition rate *R*_eff,CIS_. For the second series of samples, CIS films on uncoated soda-lime glass (SLG) served as witness samples for co-deposited layers on Mo-coated glass used in the fabrication of solar cells. This series of samples was deposited using trial and error adjustments of *T*_Cu_ and *T*_In_ together in an attempt to ensure *y* = 0.90 ± 0.03, and a range of deposition rates from ~3 Å/s to ~7 Å/s. For both series of samples, EDS was performed to determine the composition ratio *y*.

Inverted forms of Equations (4) and (5) must be applied in order to obtain predictions of *R*_eff,CIS_ and *y* for comparison with the measured values as a quantitative evaluation of the starting point of the source calibration. These forms express (*R*_eff,CIS_, *y*) in terms of the source temperatures (*T*_In_, *T*_Cu_), but require in advance the expression of the two calibration rates *R*_eff,*x*,*m*_ and fluxes *F_x_* (*x*: In, *m*: In_2_Se_3_; *x*: Cu, *m*: Cu) as polynomial functions of the source temperatures with *n*^th^ order polynomial coefficients, now denoted *a_m_*_,*n*_ and *b_m_*_,*n*_, respectively. Thus, *a_m_*_,*n*_ are the polynomial coefficients of *R*_eff,*x*,*m*_ in Figure 4, given in Table 3. The coefficients *b_m_*_,*n*_ of *F_x_*, as determined from the growth of material *m*, are given by *b_m_*_,*n*_ = (1 − *f_v,m_*)*n_x_*_,c-*m*_ *a_m_*_,*n*_ and are also presented in Table 3, based on the initial assignment of *f_v_*_,*m*_ = 0. The In or Cu fluxes described as the polynomials from Table 3 are expressed in terms of the product of the In or Cu atom concentration in CIS and the CIS effective thickness deposition rate, i.e., *F_x_* = (1 − *f_v_*_,CIS_) *n_x_*_,c-CIS_ *R*_eff,CIS_ (*x*: In, Cu). This leads to the two expressions:

(6) $$\begin{array}{cc} R_{\mathrm{eff},\mathrm{CIS}} & =\frac{1}{\left(1-f_{v,\mathrm{CIS}}\right)n_{\mathrm{In},c\text{-}\mathrm{CIS}}}\overset{2}{\underset{n=0}{\sum }}\left(1-f_{v,{\mathrm{In}}_{2}{\mathrm{Se}}_{3}}\right)n_{\mathrm{In},c\text{-}{\mathrm{In}}_{2}{\mathrm{Se}}_{3}}a_{{\mathrm{In}}_{2}{\mathrm{Se}}_{3},n}{T_{\mathrm{In}}}^{n}\\ & =\frac{1}{\left(1-f_{v,\mathrm{CIS}}\right)n_{\mathrm{In},c\text{-}\mathrm{CIS}}}\sum_{n=0}^{2}b_{{\mathrm{In}}_{2}{\mathrm{Se}}_{3},n}{T_{\mathrm{In}}}^{n} \end{array}$$

(7) $$\begin{array}{cc} y & =\frac{1}{\left(1-f_{v,\mathrm{CIS}}\right)n_{\mathrm{In},c\text{-}\mathrm{CIS}}R_{\mathrm{eff},\mathrm{CIS}}}\overset{3}{\underset{n=0}{\sum }}\left(1-f_{v,\mathrm{Cu}}\right)n_{\mathrm{Cu},c\text{-}\mathrm{Cu}}a_{\mathrm{Cu},n}{T_{\mathrm{Cu}}}^{n}\\ & =\frac{1}{\left(1-f_{v,\mathrm{CIS}}\right)n_{\mathrm{In},c\text{-}\mathrm{CIS}}R_{\mathrm{eff},\mathrm{CIS}}}\sum_{n=0}^{3}b_{\mathrm{Cu},n}{T_{\mathrm{Cu}}}^{n}, \end{array}$$

where *n*_Cu,c-CIS_ = *y n*_In,c-CIS_ has been applied in the derivation of Equation (7).

**Figure 6:**
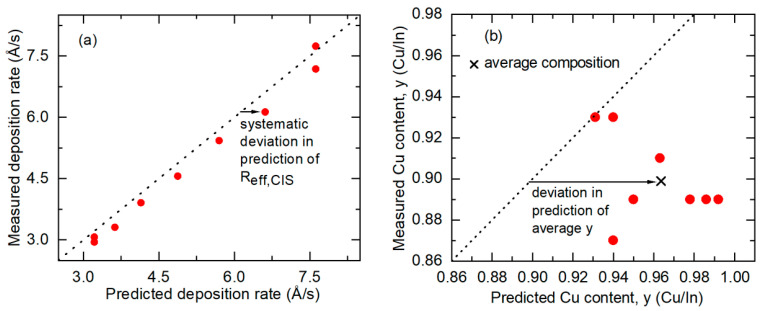
Measured CIS (**a**) effective thickness deposition rate *R*_eff,CIS_ and (**b**) [Cu]/[In] atomic ratio *y* plotted as functions of the predicted values for the CIS layers of Figure 5 with 0.87 ≤ *y* ≤ 0.93. The predicted values are identified based on the two evaporation source temperatures used in the depositions. The deviations between the measurements and predictions can be assigned to variations in the Cu and In atomic concentrations from those assumed in the calibration prediction of Figure 5.

Figure 6 summarizes the key results from Equations (6) and (7) in plots of the measured versus predicted *R*_eff,CIS_ and *y* values along with broken lines describing measurements that match predictions. The set of samples are those of Figure 5 with 0.87 ≤ *y* ≤ 0.93. Figure 6a shows that the predicted value of *R*_eff,CIS_ must be reduced by a factor of ~0.97 for improved agreement with the measurement. Based on an inspection of Equation (6), an artificially elevated prediction rate could be attributed to two possible deviations from the assumptions. First, the In atom concentrations in the In_2_Se_3_ calibration films may be lower than the single-crystal value *n*_In,c-In2Se3_ of Table 1. This corresponds to a non-zero positive value of *f_v_*_,In2Se3_ in Equation (6). Second, the In atom concentrations in the deposited CIS may be higher than the assumed single-crystal value *n*_In,c-CIS_ of Table 1. This latter possibility may occur due either to compressive stress in the CIS films or to an alternative defect model, for example one in which an additional In atom at a Cu site acts to compensate a Cu vacancy pair [30,31]. The latter defect model would lead to a higher value of *n*_In,c-CIS_ by a factor of 1.03 for CIS with *y* ~ 0.9 and a lower predicted *R*_eff,CIS_ by a factor of 0.975, relative to those of the assumed simple defect model based on Cu vacancies. Thus, improved agreement with experiment is possible with the alternative model. Section 4.1 will include further discussion of the impact of CIS defect models on calibration.

Figure 6b reveals that the prediction for the average Cu content *y* must be reduced by a factor of 0.93 in order to match the measured value. This second observation could be attributed to two possible effects as well, given that *y* can be determined as the ratio of the Cu-to-In atom fluxes. First, lower Cu concentrations may be present in the Cu calibration films than those determined by the Drude analysis used to modify the Cu concentrations from the single-crystal concentration *n*_Cu,c-Cu_ of Table 1, as shown in Figure 3a. This implies that *f_v_*_,Cu_ in Equation (7) would be greater than zero. Second, a higher In concentration may be present in the In_2_Se_3_ calibration films in comparison to the single-crystal value *n*_In,c-In2Se3_ of Table 1, resulting in *f_v_*_,In2Se3_ being less than zero in the In flux summation on the right side of Equation (6). In terms of Equation (7), the resulting increased value of *R*_eff,CIS_ from Equation (6) is in the denominator on the right side, and the predicted value of *y* is reduced. This second effect could be the result of compressive stress in the In_2_Se_3_ calibration thin films.

### 3.4. Atomic Concentration Corrections in Source Calibration for CIS Deposition

Correction factors for atomic concentration can be applied to bring the predicted CIS rates and compositions into agreement with the measured values in Figure 6 for correction of the calibration curves of Figure 5. These correction factors are simplest to develop by replacing the calibration film concentrations *n*_Cu,c-Cu_ and *n*_In,c-In2Se3_ from Table 1 by corrected values of *f*_Cu_ *n*_Cu,c-Cu_ and *f*_In2Se3_ *n*_In,c-In2Se3_, while continuing to assume that CIS has single-crystal density with only Cu vacancies that account for the variations in *y* (*y* < 1). In these corrections, *f_m_* = 1 − *f_v_*_,*m*_ is the volume fraction of material *m* with crystalline density, where *m* represents either Cu or In_2_Se_3_, and *f_v_*_,*m*_ represents the volume fraction of void space, e.g., between crystallites in the polycrystalline films. Such corrections may also account for tensile or compressive stresses in the calibration films, or deviations in the thermal expansion coefficients of the In_2_Se_3_ from values in the literature. Thermal expansion coefficients are needed because the In_2_Se_3_ calibration films are deposited at 570 °C. Finally, it should be emphasized that these atomic concentration corrections may also account for other deviations from the assumptions, including an elevated In concentration in CIS, as described in the second-from-last paragraph of Section 3.3. This deviation would lead to CIS effective thickness rates that depend not only on the temperature of the In source, as in Figure 5, but also weakly on that of the Cu source, and thus would be more difficult to implement. 

Adopting the concentration correction factors in calibration, as described in the previous paragraph, estimates can be made for their values according to:

(8) $$f_{\mathrm{Cu}}=\frac{y^{\exp}}{y^{\mathrm{th}}}f_{{\mathrm{In}}_{2}{\mathrm{Se}}_{3}}$$

(9) $$f_{{\mathrm{In}}_{2}{\mathrm{Se}}_{3}}=\frac{R_{\mathrm{eff},\mathrm{CIS}}^{\exp}}{R_{\mathrm{eff},\mathrm{CIS}}^{\mathrm{th}}}$$

In these equations, the superscript “exp” represents the experimentally determined rates and composition ratios, whereas the subscript “th” represents the associated predictions in Figure 6, based on the calibration shown in Figure 5.

**Figure 7:**
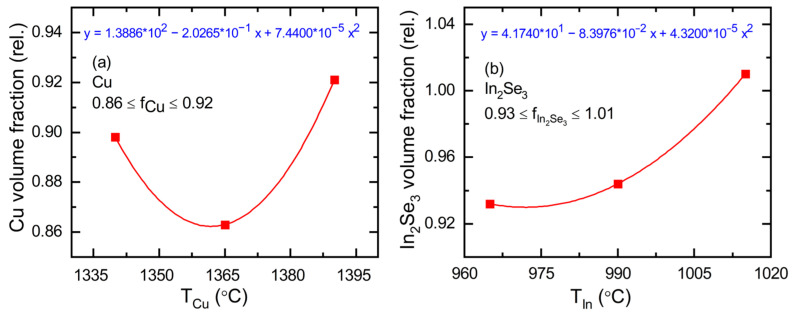
Atomic concentration correction factors *f_m_* = 1 − *f_v_*_,*m*_, where *f_v_*_,*m*_ represents the volume fraction of void, plotted as functions of the evaporation source temperatures for the (**a**) *m* = Cu and (**b**) *m* = In_2_Se_3_ calibration depositions used to develop Figure 8. The factors assigned to Cu are measured relative to Cu, with void fractions in the range 0.112–0.152 implicitly included in the effective thickness rate, as indicated in Figure 3a, and the factors assigned to In are measured relative to In_2_Se_3_ of single-crystal density.

Applying Equations (8) and (9) to the data of Figure 6, the average values of *f*_Cu_ = 0.899 ± 0.045 and *f*_In2Se3_ = 0.966 ± 0.057 are obtained. The confidence limits represent the standard deviations from the average. Systematic variations in these corrections appear to occur with the In and Cu source temperatures. These may arise either due to variations in void fraction with source temperature for the calibration films or due to a more complicated interpretation of the correction factors *f_m_* than is given by Equations (8) and (9). To quantify these variations, the two source temperatures are divided into three ranges. Average *f_m_* corrections are identified for each range and assigned to selected temperature values within the ranges. Second-order polynomials of the volume fractions versus the two source temperatures that exactly match these material volume fractions at the selected temperature values are then defined. The polynomial plots, along with their expressions, are presented in Figure 7.

**Figure 8:**
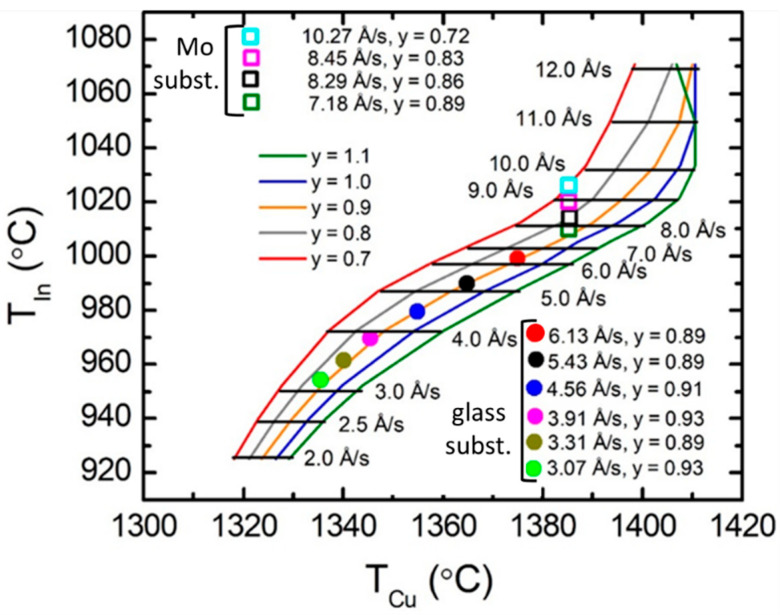
Calibration curves for Cu composition ratio *y* (curves) and CuInSe_2_ deposition rate *R*_eff,CIS_ (horizontal lines) for CIS, calculated based on the settings of the In and Cu source temperatures using the Cu and In_2_Se_3_ calibration depositions of Figure 3, modified by the atomic concentration corrections of Figure 7. Experimental results for comparison are included from two series of depositions, one series of CIS films of different *y* (open squares) deposited on Mo-coated glass and a second series at different deposition rates *R*_eff,CIS_ with intended *y* = 0.90 as witness samples deposited directly on glass for the absorber layer of solar cells (solid circles). Calibration calculations are based on the assumptions of Cu with void fractions in the range 0.112–0.152, further modified by the corrections in Figure 7a, In_2_Se_3_ with material fractions in Figure 7b, and CIS of single-crystal atomic concentrations but with Cu vacancies.

The polynomial coefficients in Figure 7 that describe the concentration correction factors can be used to generate a corrected calibration plot analogous to that of Figure 5. The construction of this corrected plot applies Equations (4) and (5). Rather than setting *f_v_*_,Cu_ and *f_v_*_,In2Se3_ to zero in those equations, yielding unity for the concentration corrections of 1 − *f_v_*_,*m*_, as for the curves of Figure 5, the corrections 1 − *f_v_*_,*m*_ = *f_m_*(*T_x_*_,0_) with *m* = Cu, In_2_Se_3_, given in Figure 7, are used. These corrections imply that the Cu and In atomic concentrations are lower than the modified values for Cu, based on the Drude analysis, and the single-crystal value for In_2_Se_3_, respectively, as applied in Figure 5. The dependence of the concentration correction *f_m_* on the source temperature, as in Figure 7, is emphasized through the notation *f_m_*(*T_x_*_,0_), where *T_x_*_,0_ are the temperature values obtained from Equations (4) and (5) prior to the atomic concentration corrections. With new evaluations of Equations (4) and (5) including these concentration corrections, much closer agreement between the calibration predictions and the experimental results can be obtained, particularly for the series of CIS layers fabricated with 0.87 ≤ *y* ≤ 0.93, as shown in Figure 8. These results track along the *y* = 0.90 line with experimental and predicted deposition rates that are in good agreement. 

**Figure 9:**
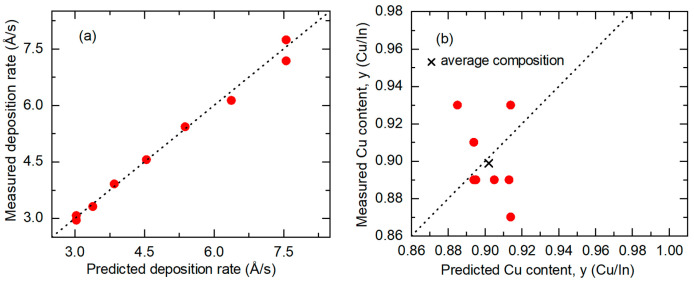
Measured CIS (**a**) effective thickness deposition rate *R*_eff,CIS_ and (**b**) [Cu]/[In] atomic ratio *y* plotted as functions of the predicted values for the deposited CIS layers of Figure 8 with 0.87 ≤ *y* ≤ 0.93. The error bar associated with the measured *y* value is ±0.03. The predicted values are identified according to the two evaporation source temperatures using the atomic concentration corrections of Figure 7. The observed root mean square deviations are reduced compared to those of Figure 6.

The new predictions incorporating the atomic concentration corrections described in Figure 7 are finally used to close the loop and update the results of Figure 6. For these new predictions, the source temperature settings can be used to define modified Cu and In atomic concentrations in the Cu and In_2_Se_3_ calibration films, respectively, from the corrections of Figure 7. Then, new predictions are obtained from Equations (6) and (7), but with the modified Cu and In_2_Se_3_ concentrations. These results are depicted graphically in Figure 9, which shows both the measured *R*_eff,CIS_ in part (a) and the measured *y* in part (b), each as a function of the predicted value for the sample series of Figure 6 with 0.87 ≤ *y* ≤ 0.93. For the effective thickness rate *R*_eff,CIS_ in Figure 9a, the measured values conform more closely to the predictions, in comparison to the corresponding results in Figure 6a obtained before the concentration corrections. For the Cu composition ratio *y* in Figure 9b, the points are clustered around the desired linear relation with measured values that are within ±0.05 of the predicted values and with an average that matches the prediction, in contrast to the results in Figure 6b. With the concentration corrections, the root mean square (rms) deviations between measured and predicted values decrease considerably, from 0.31 Å/s to 0.17 Å/s for *R*_eff,CIS_, and from 0.074 to 0.025 for *y*. The rms deviation of 0.025 from *y* is consistent with the estimated measurement error of EDS of ±0.03. 

### 3.5. Structural and Photoelectronic Properties for CIS Films Deposited at Different Rates

The trends in the structural and photoelectronic properties as functions of the effective thickness deposition rate for CIS samples with *y* ≈ 0.9 are described in this section and discussed in Section 4.2 in terms of the deposition rate reduction from the 7.6 Å/s rate predicted at the highest pair of source temperatures (*T*_Cu_, *T*_In_) = (1385 °C, 1010 °C) explored in this study. The Cu and In source temperatures yielding this rate are also the standard values used previously for CuIn_1−*x*_Ga*_x_*Se_2_ absorbers, with *x* ≈ 0.3 deposited in the same system and incorporated into solar cells with efficiencies as high as 17.4% [24].

**Figure 10:**
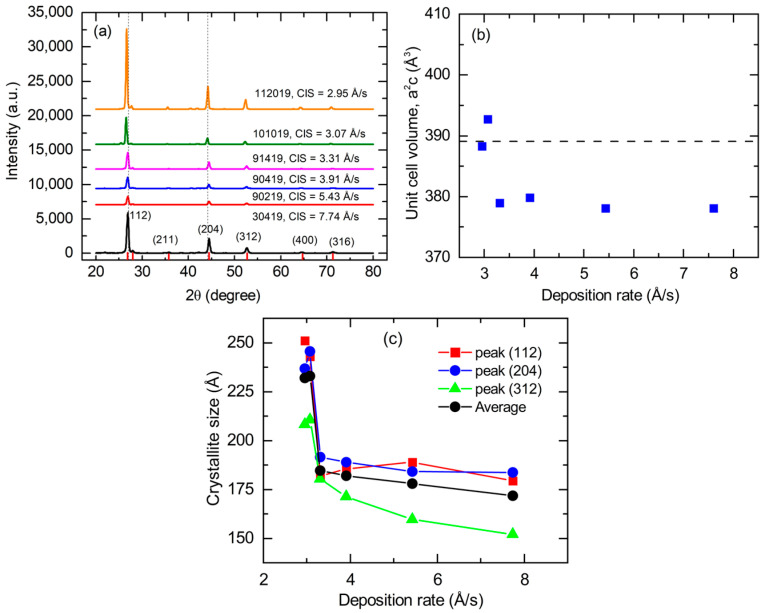
(**a**) X-ray diffraction (XRD) patterns with deduced (**b**) crystallographic unit cell volumes and (**c**) crystallite sizes for a series of CIS thin films deposited at different rates with *y* = 0.90 ± 0.03 at a substrate temperature of 570 °C. These films were deposited on Mo-coated soda-lime glass as co-deposited samples from the rate series of absorber layers in Figure 8 and Figure 9. In (**c**), Scherrer’s equation was applied independently to the first three XRD peaks representing diffractions from the (112), (220)/(204), and (312)/(116) crystal planes, and an average was also taken (black line). Parts (**a**,**c**) are reproduced from [20] with permission, 2021, IEEE PVSC.

For structural analysis, X-ray diffraction (XRD) measurements were performed on CIS films deposited at different rates in the photovoltaic device configuration on SLG substrates coated with Mo. The samples studied by XRD were co-deposited with the deposition rate series of Figure 8 and Figure 9. Thus, the source temperatures were set on the basis of trial and error, and those samples within the desired range in *y* of 0.87–0.93 were selected for this study. The XRD patterns for these CIS films are presented in Figure 10a and show close agreement with expectations for the tetragonal crystal system and chalcopyrite structure of copper (I) indium selenide [32]. The lattice parameters *a* and *c* of the films fabricated at different rates were determined from the visible (112), (211), (220)/(204), and (400)/(008) diffractions. Figure 10b shows the unit-cell volume *a*^2^*c* as a function of the CIS effective thickness deposition rate along with that for single-crystal CIS (horizontal line) [27]. The trends in the unit-cell volume, which exhibits a reduction relative to the single crystal at all deposition rates except the two lowest values (*R*_eff,CIS_ < 3.1 Å/s), are reflected in the behaviors of both lattice parameters *a* and *c*. These reductions may occur due to the deviations of *y* from the value of unity for the single crystal, or due to compressive stress in the thin films. For all samples of the series, the lattice parameter ratio of *c*/*a* = 2.000 ± 0.003 is closer to the undistorted value of 2 than to the single-crystal value of 2.014, indicating reduced tetragonal distortion in these thin films compared to the crystal.

The average crystallite size for each sample was calculated by applying Scherrer’s equation to the full widths at half maxima (FWHMs) of the three strong, lowest angle peaks (112), (220)/(204), and (312)/(116). The crystallite sizes determined independently from these FWHMs, along with the average of the resulting crystallite sizes, are plotted in Figure 10c. The average crystallite size is observed to increase gradually as the measured effective thickness rate decreases from 7.7 Å/s to 3.3 Å/s. For the two samples deposited at rates below 3.1 Å/s, the crystallite size is considerably larger, reaching its maximum value of ~230 Å over the explored range of rates. This abrupt increase in crystallite size with the rate reduction may be accompanied by a relaxation of compressive stress, a possible interpretation of the behavior in Figure 10b.

To explore the photoelectronic properties of these materials, a second series of ~2 μm-thick CIS films was prepared at different effective thickness rates with *y* = 0.90 ± 0.03, co-deposited directly on both c-Si wafer and SLG substrates at a temperature of 570 °C. This series of samples was deposited using Cu and In source temperatures determined from the calibration of Figure 8, which was corrected for atomic concentration. Ex-situ spectroscopic ellipsometry (SE) was performed on the samples to extract the bandgap and Urbach tail slope from the complex dielectric function ε over the photon energy range from 0.74 to 1.25 eV, which was centered near the bandgap of the CIS films. The results described in detail in this section were obtained from the samples deposited on c-Si, which led to smoother ε spectra upon inversion of the ellipsometry spectra (ψ, Δ), with reduced film structure-related artifacts near the bandgap. The observed trend in the Urbach tail slope parameter *E_u_* with effective thickness rate for the absorbers on c-Si was supported by the available through-the-glass SE (TG-SE) analyses of the CIS films co-deposited on the SLG substrates. 

**Figure 11:**
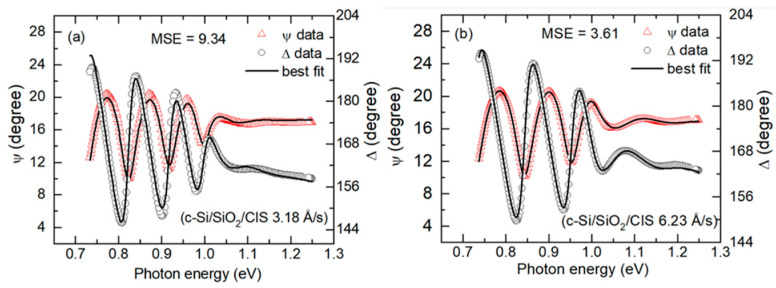
Ellipsometry angles (ψ, Δ) measured ex-situ over the photon energy range from 0.74 to 1.25 eV, along with their best fits used to determine the bandgap and Urbach tail slope for two ~2 μm-thick CIS films with measured effective thickness deposition rates of (**a**) 3.18 Å/s and (**b**) 6.23 Å/s and *y* = 0.90 ± 0.03. These samples were deposited on crystalline silicon wafer substrates at a temperature of 570 °C.

This description of the procedure for bandgap and Urbach tail analyses will use as examples the CIS films deposited on c-Si at the two intended effective thickness rates of 3.3 Å/s and 6 Å/s, corresponding to measured rates of ~3.2 Å/s and ~6.2 Å/s, respectively. Figure 11 depicts the (ψ, Δ) spectra for these two films, as obtained by ex-situ SE, along with their best fits over the photon energy range from 0.74 to 1.25 eV. These best fits utilize a structural model consisting of c-Si/SiO_2_/(CIS bulk)/(CIS roughness), where the CIS roughness layer is modeled using a complex ε spectrum determined by the Bruggeman effective medium approximation as a mixture of bulk layer CIS and void [21]. Thus, the variable structural parameters in the analysis include the bulk and surface roughness layer thicknesses and the roughness layer void content. The optical model for the bulk CIS layer includes a constant contribution to the real part of ε, denoted ε_1*o*_, one dominant critical point (CP) oscillator with a resonance energy *E*_0_ = *E_g_*, the bandgap, and a weaker Tauc–Lorentz oscillator [17]. The variable optical parameters include ε_1*o*_, *E_g_*, the CP amplitude, and the CP broadening parameter. Other optical parameters were fixed at optimized values for the complete set of samples. 

**Figure 12:**
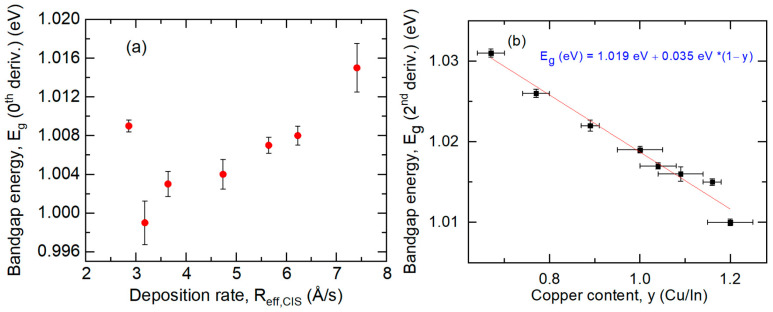
(**a**) Room temperature bandgap deduced from (ψ, Δ) spectra such as those of Figure 11 plotted as a function of effective thickness rate for CIS films with *y* = 0.90 ± 0.03 deposited on crystalline silicon wafer substrates at 570 °C. (**b**) The bandgap is also shown as a function of the CIS composition ratio *y* from mapping SE for a ~600 Å-thick, non-uniform CIS sample deposited at a rate of 7.6 Å/s from Reference [33]. Part (**b**) is reproduced from [33] with permission, 2018, IEEE WCPEC.

Figure 12a shows the resulting bandgap *E_g_* and its confidence limits for the complete set of CIS films. The bandgap is observed to decrease gradually by ~0.015 eV upon reduction of the deposition rate with a more abrupt increase at the lowest rate. Shown for comparison in Figure 12b is the bandgap plotted versus composition *y* reported earlier, obtained by mapping SE for a compositionally non-uniform CIS sample deposited at a rate of 7.6 Å/s to a thickness of 600 Å on c-Si at 570 °C [33]. Although the thicknesses of the films in Figure 12a,b differ considerably, the bandgap for the thin sample, *E_g_* = 1.022 ± 0.001 eV, according to the equation in Figure 12b with *y* = 0.90 ± 0.03, is In reasonable agreement with that for the thick sample *E_g_* = 1.015 ± 0.003 eV in Figure 12a, with the same intended rate of 7.6 Å/s. 

**Figure 13:**
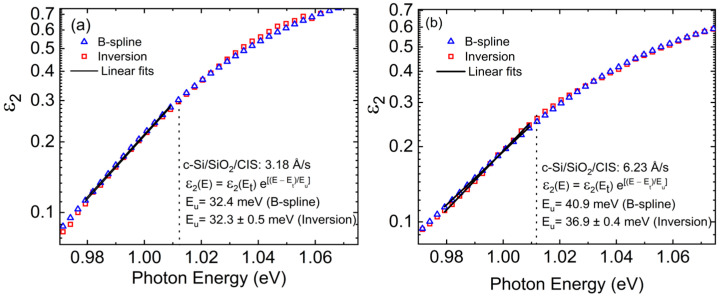
Imaginary parts of the complex dielectric functions ε_2_ plotted logarithmically versus photon energy for the CIS films deposited at (**a**) 3.18 Å/s and (**b**) 6.23 Å/s from the analysis of Figure 11. One ε_2_ spectrum was obtained by inversion (red squares) using fixed structural parameters, deduced assuming an analytical model for ε_2_, and another by Kramers–Kronig consistent B-spline smoothing (blue triangles) of the inverted results for ε_1_ and ε_2_. Shown are the fits (solid lines) to determine the Urbach tail slopes for the two versions of ε_2_, as indicated.

After achieving the best fits as shown by the illustative results in Figure 11, the deduced structural parameters were fixed, and ε was obtained by inversion of the (ψ, Δ) spectra, photon energy-by-energy. The form of the imaginary part of the complex dielectric function ε_2_ is consistent with the Urbach expression, ε_2_(*E*) = ε_2_(*E_t_*) exp{(*E* − *E_t_*)/*E_u_*}, as indicated by linearity on a logarithmic plot of ε_2_ versus photon energy *E* for *E* < *E_t_*, as in Figure 13. The slope parameter *E_u_* was obtained over the narrow range of *E* spanning ~0.03 eV below the fixed value of 1.012 eV, ~0.005 eV above the average for *E_g_* (vertical broken lines in Figure 13). As an alternative approach, the inverted ε spectra over the range from 0.74 to 1.25 eV were also fit by a Kramers–Kronig consistent B-spline model, with the results over the same narrow range also presented in Figure 13. The inversion and B-spline results can differ since the latter involves fitting both ε_1_ and ε_2_, however, the difference in Urbach slope parameters *E_u_* by the two methods is no more than 4 meV. Ex-situ TG-SE measurements were performed as well on the same set of ~2 μm-thick CIS layers co-deposited directly on SLG. Such measurements were analyzed using the same structural and optical models for the CIS films as those for films on c-Si wafer substrates. The TG-SE analysis procedure was more challenging due to the greater susceptibility of the deduced ε spectrum to artifacts and the need to account for stress in the glass.

**Figure 14:**
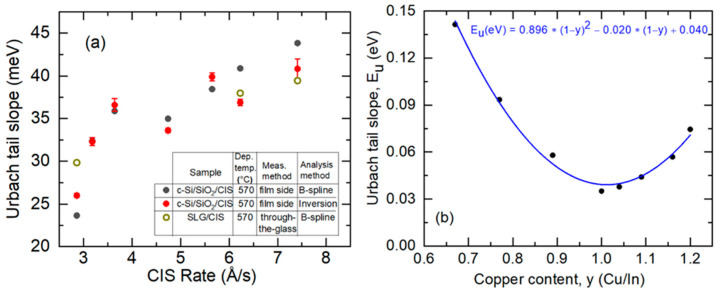
(**a**) Urbach tail slope parameters deduced from the imaginary parts of the dielectric functions ε_2_ plotted as functions of deposition rate for CIS films with *y* = 0.90 ± 0.03 deposited at 570 °C. The *ε*_2_ spectra were obtained in different ways, including by inversion using fixed structural parameters (red-filled circles) and by B-spline smoothing of the inverted result (black-filled circles), both for films on c-Si wafer substrates. In addition, results obtained from ε_2_ deduced by through-the-glass SE for CIS films on soda-lime glass substrates are included (open circles). (**b**) Urbach tail slope parameter as a function of composition ratio *y* from mapping SE for a 600 Å-thick, non-uniform CIS sample deposited at a rate of 7.6 Å/s from Reference [33]. Part (**b**) is reproduced from [33] with permission, 2018, IEEE WCPEC.

The Urbach slope parameters *E_u_* from these analyses for the complete set of samples are presented versus the effective thickness deposition rate in Figure 14a. Starting from the CIS deposited at the intended rate of ~7.6 Å/s with an Urbach slope parameter of ~40 meV, the results in Figure 14a show a gradual reduction in this parameter, with a reduction in deposition rate. A faster reduction appears to occur for rates below 3.3 Å/s, reaching a slope parameter as low as ~25 meV at the rate of ~3.0 Å/s. The TG-SE measurement and analysis were successful for three of the samples, as indicated by the open circles in Figure 14a. The trend with the CIS deposition rate in the TG-SE results is consistent with the film side results, the latter for CIS on c-Si substrates, and this further supports the conclusion of a reduction in Urbach tail slope parameter, i.e., a steepening of the Urbach tail, for reduced CIS deposition rates. The full dataset of Figure 14a is observed to follow a trend opposing, but consistent with, that of the crystallite size in Figure 10c, as will be discussed in Section 4.2.

Figure 14b shows the Urbach tail slope parameter, companion data to the results in Figure 14b, reported earlier as obtained by mapping SE for the compositionally non-uniform CIS sample deposited at a rate of 7.6 Å/s and at a thickness of 600 Å on c-Si at a substrate temperature of 570 °C [33]. The trend in Figure 14b suggests an Urbach tail slope parameter of 47 meV for *y* = 0.90, greater than the minimum of 40 meV for the stoichiometric point where disorder and potential fluctuations are minimized. This result is in reasonable agreement with the 39–44 meV range of values for the 2 μm-thick CIS in Figure 14a with *y* = 0.90 ± 0.03, and deposited at a similar rate of 7.6 Å/s. The somewhat lower slope parameter, i.e., steeper slope, in Figure 14a may be attributed to the much greater CIS thickness, which in turn may result in a larger crystalline grain size.

## 4. Discussion

### 4.1. Calibration of CIS Deposition

In the multi-source co-evaporation of CIS thin films deposited in a single-stage process, the deposition parameters of potential interest for material optimization include the atomic flux ratios, substrate temperature, and deposition rate. In studies of CIS deposited at substrate temperatures from 200 °C to 570 °C with otherwise fixed parameters, a nearly constant deposition rate was observed [34]. This suggests that all metal atoms incident on the substrate/film surface are incorporated within the film. As a result, the substrate temperature can be varied for a given pair of metal source temperatures, and the composition of the film in terms of *y* = [Cu]/[In] will remain fixed. For CIS deposition rate variations, however, the pair of metal source temperatures must be reset in such a way as to maintain the constant desired flux ratio, and thus composition ratio *y*, for the resulting films. The calibration curves of Figure 8 obtained in this study are useful for providing the source temperatures that ensure fixed *y* for any given deposition rate from ~3 Å/s to ~8 Å/s. 

In the development of these calibration curves, the first step requires identification of the source temperatures needed to generate specified fluxes of the individual elements of Cu and In, and the second step requires determination of the required fluxes to generate a CIS film of a specified rate and composition ratio. The relations developed in the first step depend on the atomic concentrations in the Cu and In_2_Se_3_ calibration films studied here, and those developed in the second step depend on the atomic concentrations in the CIS film. Thus, in establishing the calibration curves for CIS, these atomic concentration factors appear in polynomial coefficients of the form

(10) $$g_{x,\mathrm{CIS},n}={\left\{\frac{\left(1-f_{v,\mathrm{CIS}}\right)n_{x,c\text{-}\mathrm{CIS}}}{\left(1-f_{v,m}\right)n_{x,c\text{-}m}}\right\}}^{n}c_{m,n}$$

that in turn appear in Equations (4) and (5). These latter equations express the source temperatures as polynomial functions of *yR*_eff,CIS_ for the Cu source, and *R*_eff,CIS_ for the In source. In Equation (10), *c_m_*_,*n*_ together represent the polynomial coefficients that provide the source temperature of element *x* in the deposition of the calibration film material *m* in terms of the effective thickness rate *R*_eff,*x*,*m*_ of that material. The factor *f_m_* = 1 – *f_v_*_,*m*_ for the calibration film *m* in the denominator of Equation (10) is adjusted based on the results of Figure 7 so that the predicted and measured CIS deposition rate and composition are in close agreement. The reduced volume of the unit cell observed for thin film CIS compared to the single crystal, however, as shown in Figure 10b for all rates but the lowest, impact these adjustments. This effect would lead to values of 1 – *f_v_*_,CIS_ ≈ 1.03 for all but the lowest rates, and necessitate correspondingly reduced values for *f_v_*_,Cu_ and *f*_*v*,In2Se3_ in Figure 7 to compensate. Because of the uncertainties related to the concentration of Cu and In in the deposited CIS thin films, depending on the composition, the film structure and stress, and in particular the appropriate defect model as described in Section 3.3 and further in the next paragraph, the concentration corrections were assigned to the Cu and In_2_Se_3_ calibration films.

The approach applied as described In Section 3.4 is based on atomic fractions of Cu, In, and Se in CIS of *y*/(*y* + 3), 1/(*y* + 3), and 2/(*y* + 3), and concentrations of 4*y*/*a*^2^*c*, 4/*a*^2^*c*, and 8/*a*^2^*c*, respectively, thus assuming no change in the crystal structure with *y*. Given that each Cu vacancy would give rise to a free hole, however, this model for the defect structure cannot account for the low hole concentrations in photovoltaic device quality materials [12]. For the CIS samples in this study deposited on SLG with *R*_eff,CIS_ = 3.1 Å/s and 7.6 Å/s, and with *y* ~ 0.90, the hole concentration was 5 × 10^16^ cm^−3^, as measured by terahertz SE. This hole concentration, determined assuming an effective mass of 0.73*m_e_*, where *m_e_* is the free electron mass, was a factor of 10^2^ lower than that in an earlier investigation [29], likely due to CIS material improvements over time. If it is assumed instead that, for each pair of Cu vacancies, an extra compensating In is incorporated, i.e., yielding the defect complex 2V_Cu_-In_Cu_ as described in Section 3.3 [30,31], then the atomic fractions of Cu, In, and Se would be 2*y*/(3*y* + 5), 2/(3*y* + 5), and (*y* + 3)/(3*y* + 5), respectively, with concentrations of 16*y*/(*y* + 3)*a*^2^*c*, 16/(*y* + 3)*a*^2^*c*, and 8/*a*^2^*c*, again assuming no change in crystal structure with *y*.

Application of the 2V_Cu_-In_Cu_ defect model for the CIS atomic concentrations yielded improved results over the simple vacancy model when no atomic concentration corrections were assigned to the calibration films. The atomic concentration corrections must be applied when using this defect model, however, in order to reach the agreement between the prediction and experiment (rms deviations of 0.20 Å/s for *R*_eff,CIS_ and 0.035 for *y*), comparable to those of Figure 9 (0.17 Å/s and 0.025, respectively). The best fitting calibration film corrections for the 2V_Cu_-In_Cu_ defect model showed the same trends as in Figure 7 but spanned the ranges from a minimum of *f*_Cu_ = 0.90 to a maximum of *f*_Cu_ = 0.97 for the Cu source calibration (as compared to the range of 0.86–0.92 for the Cu vacancy model of Figure 7), and from a minimum of *f*_In2Se3_ = 0.95 to a maximum of *f*_In2Se3_ = 1.06 for the In source calibration (as compared to the range of 0.93–1.01 in Figure 7). Thus, for this defect model to be valid, it must be concluded that In_2_Se_3_ deposited at the higher rates is under compressive stress, having as much as a 6% higher In atom concentration than the single crystal. Finally, given that the unit cell volume of CIS is weakly varying with *R*_eff,CIS_, increasing only at the lowest rates, and considering that the defect model is unlikely to change as *R*_eff,CIS_ decreases, one must conclude that the key to accurate calibration is correction for the atomic concentrations of the calibration films, as in Figure 7. 

**Figure 15:**
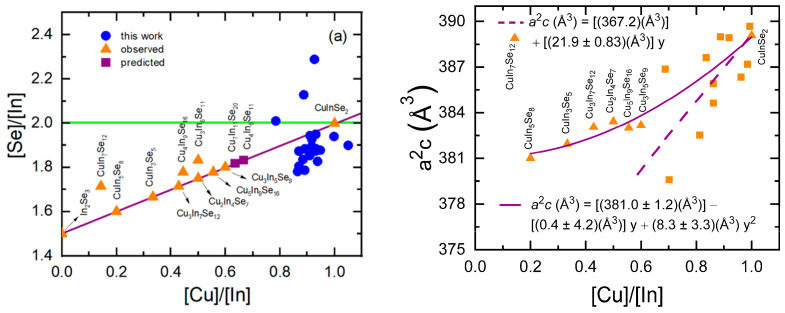
(**a**) Relationship between the [Se]/[In] atomic ratio and the [Cu]/[In] atomic ratio for defect models consisting of Cu vacancies without (green line) and with compensating defects (violet line). The compensating defect model 2V_Cu_-In_Cu_ is assumed here along with the associated predicted (squares) and observed (triangles) phases [31,32]. Experimental data collected in the present study are also shown for CIS as absorber layers in solar cells (circles). (**b**) Unit-cell volumes from XRD studies of CIS in the literature [27] and the following ordered defect phases derived from CIS, including Cu_3_In_5_Se_9_ [39], Cu_5_In_9_Se_16_ [39], Cu_2_In_4_Se_7_ [39], Cu_3_In_7_Se_12_ [40], CuIn_3_Se_5_ [39], CuIn_5_Se_8_ [41,42], and CuIn_7_Se_12_ (triangles) [43]. Also shown are corresponding results for a collection of 11 thin-film samples with 0.6 < *y* < 1.0 (squares) [44].

To explore the possible defect models further, Figure 15a shows the atomic ratio of [Se]/[In] versus *y* = [Cu]/[In], a plot that fully characterizes the compositional deviations of the CIS films. The horizontal line at [Se]/[In] = 2 describes the model assuming uncompensated Cu vacancies, whereas the diagonal line describes the model assuming the compensating defects 2V_Cu_-In_Cu_. Thus, In_2_Se_3_ with *y* = 0 and several observed and predicted ordered defect phases lie along or near the diagonal line [30,31]. Most data values from the present study, corresponding to the full range of *y* ~ 0.85–1.05, lie below the diagonal line in Figure 15a, indicating Se-poor character. A few outlying data points, corresponding to Se-rich compositions, were obtained preferentially for the lower-rate CIS depositions. Assuming that the EDS compositions are accurate, other possible defects as described in the literature could play a role in these CIS films [35,36,37,38]. It has been suggested that CIS exhibits a strong preference for both Cu and Se vacancies over In vacancies [38]. Since it is proposed that the V_Se_-V_Cu_ divacancy can exhibit donor-like character [37] in p-type CIS, such a compensating defect could explain both the compositional trend in Figure 15a and the low hole concentration, and would yield the same calibration outcome as the simple model of uncompensated Cu vacancies assumed here. The outlying data for Se-rich CIS in Figure 15a remain interesting, however, and require further study.

Figure 15b shows the unit-cell volume of the observed ordered defect phases, whose compositions are given in Figure 15a (triangles). These results are obtained by XRD in several studies in the literature [39,40,41,42,43]. Also shown are corresponding results for a collection of thin-film CIS samples with 0.6 < *y* < 1.0 (squares) [44]. Excluding the outlying point for the lowest *y* value of 0.14, trends toward lower unit-cell volume are observed with decreasing *y* in both datasets, as indicated by the quadratic and linear fits to the first and second datasets, respectively. Applying this trend with decreasing *y* between *y* = 1.0 and *y* = 0.9, a reduction in unit-cell volume of only 0.5% relative to single-crystal CIS is predicted, a smaller deviation than is observed for all CIS samples with the exception of those deposited at the lowest effective thickness rates in Figure 10b. This suggests that the reduction in unit cell volume in Figure 10b is due to compressive stress which is relaxed at the lowest rates and largest grain sizes.

In summary, given the observed requirement of concentration corrections in the calibration depositions and their dominance in this study, as well as the uncertainties in the appropriate Cu and In concentrations in the CIS thin film, evaluation of different defect models within the calibration methodology has not been explored in detail here. As a result, the approach applied to generate the calibration curves has not been extended beyond the simple Cu vacancy model described in Section 3.4. Future prospects exist for refinement of the calibration, and through that refinement, insights into the defect structure of CIS may be obtained.

### 4.2. Effect of Deposition Rate on the Properties of CIS 

Next, the effect of deposition rate on the structural and photoelectronic properties of CIS will be discussed, focusing on the composition *y* = 0.90 ± 0.03. The trend toward increased grain size with the reduction in effective thickness rate in Figure 10c is consistent with the suggestion that crystallite growth is controlled by the diffusion of metal atoms on the surface. As the deposition rate decreases, an increase occurs in the time available for diffusion before the next monolayer is deposited. The same concept can account for the relaxation of the volumetric compressive stress that occurs at the lowest rates where the crystallite size increases most rapidly. The potential effect on the CIS properties generated by the increase in Se atom flux relative to the metal atom flux as the deposition rate decreases, however, should also be considered [45]. It is generally understood that the In_2_Se_3_ calibration film and the CIS films with different effective thickness rates studied here are both deposited under a higher flux of Se than is necessary to form the crystalline phases of these materials. As a result, the excess Se must either be released after incorporation or simply reflected from the surface of the film. The increasing excess flux of Se with decreasing In and Cu source temperatures, and CIS deposition rate, may assist in promoting grain growth of the In_2_Se_3_ and CIS layers via the generation of lower-energy bonding configurations due to the rapid exchange of Se between the solid and gaseous phases. As indicated by the outlier points in Figure 15a, the increasing relative flux of Se at the lowest rates may suppress the formation of Se vacancies and vacancy complexes [46].

The latter effects can be quantified by measuring the growth of Se on a room-temperature quartz crystal oscillator. In these studies, the Se source was set at a temperature such that the measured Se deposition rate was 20 Å/s. Assuming that all Se atoms impinging on the quartz crystal at room temperature are incorporated within the layer, and using the mass density of the resulting amorphous Se film as 4.28 g/cm^3^ [47], then the predicted Se flux is 0.653 atoms/Å^2^s. Using the atomic concentration-corrected flux of In in the formation of In_2_Se_3_ for an In source temperature that increases from 965 °C to 1025 °C in Figure 3b, then the flux of Se is predicted to decrease from 12 to 4 times that needed to form the In_2_Se_3_ thin film. Thus, at the highest In source temperature, for every four Se atoms impinging on the In_2_Se_3_ surface, three are released or reflected. In the case of the rate series of CIS depositions in Figure 8 and Figure 9 with In source temperatures increasing from 955 °C to 1025 °C, the Se flux decreases from 11 to 3 times higher than the flux needed to form a CIS film of crystalline atomic concentrations. These flux-ratio estimates assume In:Se ratios of the single crystals, with Cu vacancies in the case of CIS.

Turning to the optical properties, the results in Figure 12 will be discussed first in view of the bandgap values and their variations reported in the literature for single-crystal and polycrystalline bulk CIS. The reported bandgap values for bulk CIS materials range from 0.96 eV in the first studies [48,49] to 1.01 eV [50,51] and even as high as 1.03–1.04 eV in later works [52,53,54]. In Reference [49], it was suggested that wider bandgaps in polycrystalline films compared with bulk crystals were due to grain boundary effects. In later research [54], it was proposed that the widest bandgaps were associated with the stoichiometric CIS crystals, and that the narrower gap materials reflected deviations in stoichiometry typical of thin films, both Cu-rich and In-rich. The latter behavior may arise due to ε_2_ contributions from more extensive Urbach tails, as in Figure 14b, that shift the apparent bandgap to lower energies—trends also suggested by previous data [55]. Alternatively, the narrowing of the bandgap observed in CIS has been attributed to the effects of free carriers and ionized impurities on the screening length [50], which presumably could also account for the reported differences in bandgap. Differences in bandgap among CIS materials have been observed due to differences not only in stoichiometry and carrier concentration, but also in the in-plane stress [53]. 

The increase in bandgap with reduction in *y* reported previously [33] and reproduced in Figure 12b differs from the behavior reported in reference [53], but is consistent with the observed trends toward reduced lattice constants and unit-cell volume illustrated in Figure 15b. The reduction in the bandgap with the decrease in deposition rate from 7.5 Å/s to 3.5 Å/s in Figure 12a cannot be attributed to additional ε_2_ contributions from the Urbach tail [54], as Figure 14a shows a trend toward a reduced Urbach tail slope over this range. An increase in carrier concentration [50] also appears unlikely due to the near-constant values of ~5 × 10^16^ cm^−3^ obtained by terahertz SE over this range of rates. Possible effects to explain the observed trend include a reduction in the effect of grain boundaries [48] and/or a gradual reduction in the in-plane compressive stress [53,56], as probed by SE, both occurring when the grain size gradually increases with the rate reduction, as shown in Figure 10c. A weak reduction in compressive stress is observed in the XRD studies of Figure 10b, however, the samples of that study were SLG/Mo/CIS, which may show differences relative to SLG/CIS studied for Figure 12 and Figure 14. The rapid increase in bandgap at the lowest rate may be due to a reduction in the effects of the Urbach tail in narrowing the bandgap when the tail sharpens considerably. In fact, this material has the narrowest Urbach tail and the largest grain size among the series of samples (as discussed next), and is most likely to exhibit a bandgap close to the single crystal [54].

The trend observed in the Urbach tail slope in Figure 14a opposes that of the crystallite size in Figure 10c. This suggests that the Urbach tail slope is controlled by defect states at grain boundaries. Alternatively, or in addition, carrier scattering may occur which limits the excited state lifetime of the bandgap transition. The larger grain size would lead to a longer mean free path and mean free time of the optically excited carriers and less broadening of the bandgap transition, as reflected in the steeper Urbach slope. Assuming that large grains are associated with enhanced electronic quality for a CIS absorber layer, then the results of Figure 10c and Figure 14a would suggest improved photoelectronic quality continuously as the absorber layer deposition rate is reduced. Studies of completed solar cell devices have suggested that a poorer CdS/CIS junction achieved with the lowest-rate CIS absorbers may explain an optimum cell performance observed for a rate of 3.3 Å/s, just above the minimum rate explored both in this study and that of Reference [20]. 

## 5. Summary

A metal atom flux calibration procedure was developed and applied to CuInSe_2_ (CIS) thin films and device structures deposited by single-stage thermal co-evaporation using individual elemental sources of Cu, In, and Se. In this calibration, real-time spectroscopic ellipsometry (SE) was applied to a sequence of Cu depositions performed on a single substrate at successively increasing evaporation source temperatures. A similar procedure was applied to a series of In_2_Se_3_ depositions at successively increasing In source temperatures. The results of the calibration were curves that provided the source temperatures required for the deposition of CIS films of fixed specified composition ratio *y* = [Cu]/[In] and variable deposition rate, the latter a key parameter in any thermal co-evaporation process. Accurate calibration curves rely on atomic concentration corrections that are assigned to the calibration films. These corrections amount to deficits of a total of ~22% for Cu and ~3% for In_2_Se_3_, relative to the Cu and In_2_Se_3_ single crystals, respectively, and appear to depend on the deposition rate of the calibration films. The larger deficit for Cu may result in part from the thinness of the 40 Å Cu calibration films compared to the 2000 Å In_2_Se_3_ films. The calibration curves have been applied to deposit CIS films with fixed [Cu]/[In] ratios of *y* = 0.90 ± 0.03, and deposition rates decreasing from 8 Å/s to 3 Å/s, given in terms of effective thickness or volume per planar area, for measurement of the structural and photoelectronic properties. Improvements in these properties, as indicated by the increase in grain size deduced by X-ray diffraction and the steepening of the Urbach tail slope deduced by SE, occur with the reduction in deposition rate, with the largest improvement occurring at the lowest rate of 3 Å/s. 

Assuming the correct assignments of the atomic concentration corrections to the calibration films, accurate atomic fluxes (within ±0.5%) can be established as functions of the source temperatures. These functions will also enable the development of more advanced multiple-stage depositions in which each metallic source is operated individually. The standard three-stage process used for CuIn_1−x_Ga_x_Se_2_ (CIGS) absorber layers for solar cells, and also applied for the highest-efficiency CIS cells [57], starts from a deposition of (In_1−x_Ga_x_)_2_Se_3_ (IGS) using a flux of In, Ga, and Se, and continues with a conversion of the IGS to Cu-rich CIGS from a flux of Cu, and concludes with additional In, Ga, and Se exposure for Cu-poor CIGS [12,13,58]. For the CIS absorber deposited by this method, knowledge of the flux of Cu and In as functions of source temperature allows each stage to be performed at a different deposition or conversion rate. In fact, RTSE studies have shown that the highest-efficiency CIGS cells are fabricated starting from IGS absorbers of the largest crystallite size, which are observed for a [Ga]/{[In] + [Ga]} ratio of *x* ≈ 0.3 [24]. This suggests that improvement in the materials deposited from such three-stage processes may be possible through proportionate reductions in the Cu and In fluxes to allow for enhanced crystallite growth. Alternatively, decoupling the fluxes during the three stages, and optimizing them individually rather than adopting the same values used in the single stage process, may lead to improved materials. 

## Figures and Tables

**Table 1:** Crystallographic data for Cu, In_2_Se_3_, and CuInSe_2_ (CIS) applied in evaporation source calibration. From left to right, these data include the substrate temperature for thin film deposition of the material, its crystal structure, the lattice parameters at room temperature, the number of metal atoms per unit cell, the concentration of atoms in the crystal at room temperature, the thermal expansion coefficient, and the concentration of atoms at the deposition temperature. The references for the lattice parameters and thermal expansion coefficients are provided. For CIS, *y* is the atomic concentration ratio of Cu to In, i.e., *y* = [Cu]/[In], assumed here to be less than unity due to uncompensated Cu vacancies that do not affect the lattice parameters.

Material and Its Thin Film Deposition Temperature *T*	Crystal Structure	Lattice Parameters at 20 °C [Ref.] (Å)	Number of Metal Atoms per Unit Cell	Concentration of Metal Atoms at RT (Å^−3^)	Thermal Expansion Coefficient α(*T*) = *P*_1_ (10^−6^ K^−1^) + *P*_2_ (K)/*T*^2^ [Ref.]	Concentration of Atoms at Temperature *T* (Å^−3^)
Cu *T* = 20 °C	face-centered cubic	*a* = 3.6147 [23]	4	0.084692	*P*_1_ = 14.87 [23]	0.084692
In_2_Se_3_ *T* = 570 °C	defect wurtzite	*a* = 7.1236 *c* = 19.381 [25]	12	0.014069	*P*_1_(*a*) = 5; *P*_1_(*c*) = 16 *P*_2_(*a*) ~ 0; *P*_2_(*c*) ~ 0 [26]	0.013869
CuInSe_2_ *T* = 570 °C	chalco- pyrite	*a* = 5.7810 *c* = 11.6422 [27]	Cu: 4*y* In: 4 (assumed)	Cu: 0.010281*y* In: 0.010281	*P*_1_(*a*) = 9.15; *P*_1_(*c*) = 7.61 *P*_2_(*a*) ~ 0; *P*_2_(*c*) ~ 0 [28]	Cu: 0.010135*y* In: 0.010135

**Table 2:** Polynomial coefficients *c_m_*_,*n*_ of order *n* that describe the evaporation source temperature *T_x_* for element *x* as a function of the effective thickness rate *R*_eff,*x*,*m*_ of material *m* as in Equations (2) and (3). Also provided are the coefficients *d_m_*_,*n*_ that describe *T_x_* as a function of the flux *F_x_* during the growth of material *m* at its deposition temperature, where the flux is given as *F_x_* = *n_x_*_,c-*m*_ *R*_eff,*x*,*m*_ with *n_x_*_,c-*m*_, representing the concentration of atoms *x* in the single crystal of material *m* from Table 1. The unaccounted-for (excess or deficit) void fraction *f_v_*_,*m*_ in Equation (1) is set to zero.

Material, *m*, and Indept. Variable	*c_m_*_,0_ (°C) or *d_m_*_,0_ (°C)	*c_m_*_,1_ (°C s/Å) or *d_m_*_,1_ (°C Å^2^ s)	*c_m_*_,2_ (°C s^2^/Å^2^) or *d_m_*_,2_ (°C Å^4^ s^2^)	*c_m_*_,3_ (°C s^3^/Å^3^) or *d_m_*_,3_ (°C Å^6^ s^3^)
Cu; *R*_eff,Cu,Cu_	1.29980 × 10^3^	9.59360 × 10^1^	2.00584 × 10^1^	−2.30861 × 10^1^
Cu; *F*_Cu_	1.29980 × 10^3^	1.13276 ×10^3^	2.79648 × 10^3^	−3.80034 × 10^4^
In_2_Se_3_; *R*_eff,In,In2Se3_	8.40985 × 10^2^	6.85232 × 10^1^	−1.05532 × 10^1^	6.57389 × 10^−1^
In; *F*_In_	8.40985 × 10^2^	4.94075 × 10^3^	−5.48649 × 10^4^	2.46426 × 10^5^

**Table 3:** Polynomial coefficients *a_m_*_,*n*_ of order *n* that describe the effective thickness rate *R*_eff,*x*,*m*_ of material *m*, and the coefficients *b_m_*_,*n*_ that describe the flux *F_x_* of the associated metal atom *x*, both as functions of the evaporation source temperature for atom *x*. The relation between the coefficients is *b_m_*_,*n*_ = (1 − *f_v,m_*)*n_x_*_,c-*m*_ *a_m_*_,*n*_.

Material, *m*, and property	*a_m_*_,0_ (Å s^−1^) or *b_m_*_,0_ (Å^−2^ s^−1^)	*a_m_*_,1_ (Å s^−1^ °C^−1^) or *b_m_*_,1_ (Å^−2^ s^−1^ °C^−1^)	*a_m_*_,2_ (Å s^−1^ °C^−2^) or *b_m_*_,2_ (Å^−2^ s^−1^ °C^−2^)	*a_m_*_,3_ (Å s^−1^ °C^−3^) or *b_m_*_,3_ (Å^−2^ s^−1^ °C^−3^)
Cu; *R*_eff,Cu,Cu_	−1.54970 × 10^3^	3.44158 × 10^0^	−2.55428 × 10^−3^	6.33745 × 10^−7^
Cu; *F*_Cu_	−1.31247 × 10^2^	2.91475 ×10^−1^	−2.16327 × 10^−4^	5.36731 × 10^−8^
In_2_Se_3_; *R*_eff,In,In2Se3_	2.68050 × 10^2^	−5.96550 × 10^−1^	3.33333 × 10^−4^	--
In; *F*_In_	3.71758 × 10^0^	−8.27355 × 10^−3^	4.62300 × 10^−6^	--

## Data Availability

Data underlying the results presented in this paper are not publicly available at this time but may be obtained from the authors upon reasonable request.
